# The mediating role of core self-evaluation in the association between perceived peer relationship quality and loneliness in university students

**DOI:** 10.1371/journal.pone.0317310

**Published:** 2025-01-13

**Authors:** Hongxiu Tan, Wen Xiao

**Affiliations:** School of Educational Science, Shaoguan University, Shaoguan, Guangdong, China; Independent, AUSTRALIA

## Abstract

This study investigated the mediating role of core self-evaluation (CSE) in the relationship between the perception of peer relationship quality and loneliness among university students during the COVID-19 pandemic. An online survey was conducted with 462 university students (mean age: 20.7 ± 1.56 years; age range: 18 to 25 years) using the Peer Relationships Satisfaction Scale, Core Self-Evaluation Scale, and UCLA Loneliness Scale. The results revealed a significant negative correlation between perceived peer relationship quality and loneliness, as well as between CSE and loneliness. Core self-evaluation accounted for 36.23% of the total effect of peer relationship quality on loneliness. Additionally, female students reported higher levels of loneliness than male students, and students from rural areas experienced greater loneliness than their urban counterparts. The study concluded that fostering high-quality peer relationships and enhancing core self-evaluation could be effective strategies for reducing loneliness among university students.

## Introduction

The COVID-19 pandemic has presented an unprecedented global challenge and has had serious implications for the economy and health of many countries. Since the onset of the pandemic, many countries, including China, have implemented measures such as mandatory lockdown for the entire country, strict social and physical distancing, and mandatory closures of all schools. While these measures were imposed to slow or prevent the transmission of the COVID-19 virus, the consequences on mental health have received increasing attention [1,2]. Particularly among young people, social isolation and loneliness may result from the disruption in their daily routines and social interactions with peers and family [3].

Loneliness is a negative subjective emotional state marked by discomfort and distress arising from a gap between desired and actual social relationships, either in terms of quality (lack of close connections) or quantity (insufficient contacts) [4]. Research has shown that the government-mandated lockdown to control the spread of coronavirus may lead to feelings of isolation [5]. Studies indicate that young individuals have experienced higher rates of loneliness during the COVID-19 pandemic compared to older adults [6,7]. Specifically, between 38% and 50% of individuals aged 18–24 reported increased levels of loneliness during the enforced lockdown [5,6]. Furthermore, females were found to be more likely to experience loneliness than males [8]. Prolonged loneliness is strongly associated with various adverse mental and psychological consequences. Evidence has identified loneliness as a strong precursor of anxiety, stress, depression, and suicide. It was noted that lonely youth were three times more likely to develop depression later, with effects lasting up to nine years [9]. Given that most mental health disorders emerge in individuals in their twenties and early thirties, and considering the higher prevalence of such disorders in college and university student populations compared to the general public [10], it is crucial to address loneliness among this demographic group. This study investigates how such isolation affects the mental health of university students, focusing on loneliness, peer relationships, and core self-evaluation.

### Perceived peer relationship quality and loneliness

Loneliness is closely tied to the quality of interpersonal relationships, particularly with peers, which are essential for young individuals’ psychological well-being [11]. For young adults, interpersonal connections are crucial, with physical proximity during social interactions fostering attachment, trust, and mutual connectivity by promoting shared experiences and interactions [12]. The human brain benefits from close relationships, as these intimate bonds foster social integration and positively influence mental well-being [13]. Defined by equality and reciprocity, peer relationships refer to an individual’s perception of the quality of interactions and bonds formed with peers—those of similar age or developmental stage—through shared experiences and mutual support [13].

Perlman argued that loneliness results from a gap between an individual’s ideals and actual achievements in interpersonal relationships, with greater gaps intensifying feelings of loneliness [14]. Existing research, primarily focusing on younger populations such as elementary and middle school students, has identified an inverse relationship between the quality of peer relationships and feelings of loneliness [15,16]. However, there is a research gap in exploring this dynamic among university students, particularly under the unique circumstances of the COVID-19 pandemic. The restrictions imposed during lockdowns, such as home confinement and social distancing, may impede face-to-face interaction, which are considered essential for personal growth in early adulthood [17]. A study indicated that in-person emotional support slightly decreased the likelihood of depression, while emotional support provided through online platforms slightly increased the chances of experiencing depression [18]. Consequently, the lack of social engagement, diminished peer support, and feelings of isolation might lead to the onset of depressive symptoms among young adults during the pandemic. Given these important social changes in young adulthood, the present study aimed to investigate the correlation between the perception of the quality of peer relationships and loneliness among university students during the pandemic. We hypothesized a negative correlation between the perception of the quality of peer relationships and the experience of loneliness among this demographic group.

### The mediating role of core self-evaluation

In terms of a social-cognitive model of loneliness, hypersensitivity to social threat and social reward could result in a vicious circle in which lonely individuals perceive their environment as more negative and less positive, which in turn can lead to more negative interactions and negative affect, and finally result in even higher levels of loneliness [19]. Core self-evaluation (CSE) is a higher-order personality construct comprising four key elements: global self-esteem, generalized self-efficacy, internal locus of control, and emotional stability [20]. CSE is a universal personality trait that represents an individual’s fundamental assessment of their own abilities and self-worth. Previous studies have found that individuals with positive self-evaluations are less likely to experience psychological distress and burnout [21,22]. For example, as specific traits in CSE, self-esteem and self-efficacy have been shown to negatively correlate with loneliness [23,24]. Moreover, loneliness, an inherently subjective psychological experience, was found to be profoundly shaped by individual personality traits such as neurotism [25]. In addition, some researchers also provided evidence that loneliness are negatively related to external locus of control [26]. However, so far as we know there are few studies on the direct relaitonship between core self-evaluation and perceived loneliness and the nature of this link between the two elements remains ambiguous. It is necessaray to explore the effect of core self-evaluation on the feelings of loneliness among university students.

In addition, university students’ core self-evaluation may be influenced by their perception of the quality of peer relationships. Researchers have underscored the pivotal role of CSE in shaping life experiences, suggesting that it not only responds to but also actively influences environmental factors and the assimilation of external information [27]. The transition from high school to college seems to be a challenging period in the social lives of young adults, as they often leave their parents’ home and have to establish new social relationship, while maintaining the existing familial and peer relationships [28]. The difficulties that young adults experience with these transitions have been related to decreases in emotional well-being, such as higher levels of depressive feelings and particularly increased feelings of loneliness. Based on previous evidence, we hypothesized that perceived peer relationship quality were positively related to CSE and that CSE played a mediating role in the effect of perceived peer relationship quality on loneliness among university students.

### The current study

This study adopted the descriptive cross-sectional design to provide a comprehensive understanding of the mechanisms through which peer relationships influence university students’ perceived loneliness. Previous research has shown that loneliness among university students is associated with factors such as residence in low- or middle-income countries, family wealth, year of study, gender, academic performance, and the need for romantic relationships, social interactions, and economic support [29–31]. However, due to cultural differences, these findings may not be directly applicable to students at Chinese universities. For instance, studies in Western contexts have found that higher levels of individualism are associated with greater feelings of loneliness among university students [7]. In contrast, Chinese culture emphasizes collectivism, which may lead to different experiences and expressions of loneliness. Furthermore, a systematic review of over 20 studies on university students, primarily conducted in the USA, explored the associations between adult friendship and well-being [32]. This study also highlighted the scarcity of studies examining loneliness and social relationships among Chinese students. In addition to cultural differences, an important factor that makes this study unique is the prolonged impact of COVID-19 lockdowns in China, which lasted significantly longer than in many other countries. This extended period of confinement could have had distinct psychological effects on Chinese university students, especially regarding their experiences of loneliness and peer relationships. This unique context strengthens the rationale for the present study, highlighting the importance of understanding the long-term consequences of these extended restrictions on university students in China.

**Fig 1:**
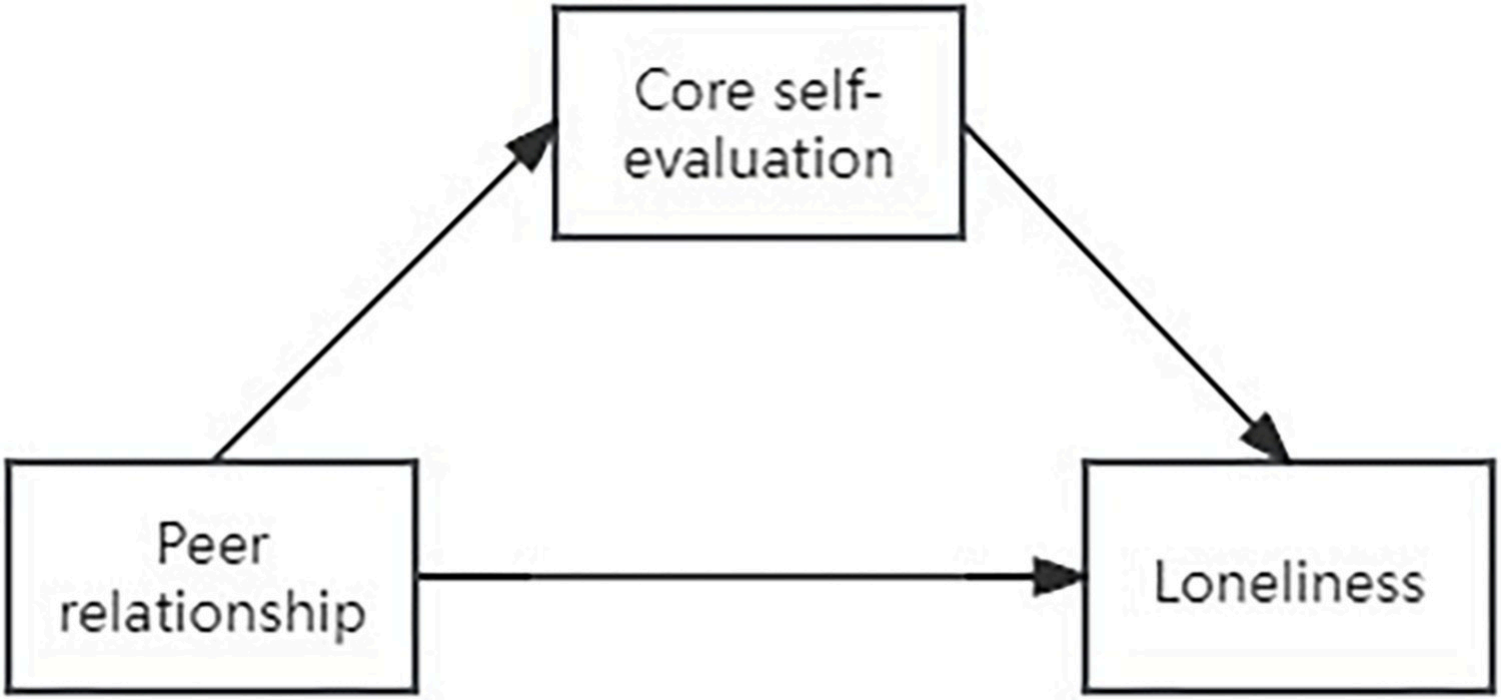
The path diagram of the model.

To address this, we compared loneliness and related variables across demographic factors such as gender, sibling status, academic year, major, and place of origin among Chinese university students. We examined whether young adults’ core self-evaluation is a potential mediator of the connection between peer relationship and loneliness (Fig 1). Through examining the influence of perceived peer relationship quality on CSE, and subsequently their impact on loneliness, this research endeavored to unravel the intricate mechanisms that underlie the prevalence of loneliness among university students. The findings would have the potential to inform targeted interventions that enhance peer connections and CSE, thereby reducing loneliness and improving well-being of university students.

## Materials and methods

### Procedure and sample

For this study, we employed a convenience sampling method, targeting participants who were readily available and willing to participate. This method was chosen due to the accessibility of the population and the constraints of the study timeframe. Data was collected over a period of two weeks in October 2022, coinciding with China’s implementation of the dynamic zero-COVID strategy. This approach aimed to curb virus spread through brief lockdowns and rigorous identification, testing, contact tracing, and isolation procedures. The online survey was conducted using the Psycloud platform, which is a widely used and secure tool for collecting research data. The study was approved by the institutional review board of the University Research Ethics Committee. Participants were recruited from a University in south China. Recruitment was conducted via a quick response (QR) code or web link for the electronic questionnaire distributed via WeChat, and announcements were made in relevant student groups and forums. Students were asked to complete the questionnaire online in a private space without any time constraints. Oral consent was obtained prior to participation. Several measures were implemented to prevent multiple submissions from the same participant. First, participants were required to log into their university credentials, which allowed us to restrict survey access to only one response per individual. Second, a survey platform was set up to track IP addresses and prevent multiple submissions from the same IP address. Finally, we included attention-check questions within the survey to ensure data quality and detect inconsistent responses.

In total, 495 university students filled in the questionnaire. After screening, incomplete responses (e.g., missing more than 20% of the items), duplicate submissions, and responses that showed signs of random answering (e.g., patterns of answering all items in the same direction or inconsistent responses to attention-check questions) were removed. Following this, 462 questionnaires were considered valid, yielding an effective response rate of approximately 93.33%. According to a previous study, a sample size of 405 participants is required to conduct mediational studies with a statistical power of 0.8 [33]. The sample size was considered appropriate for the analysis in our study. The average age of the 462 participants was 20.7±1.56 years. Table 1 presents the demographic distribution of the sample. Among them, 212 were male (45.9%) and 250 were female (54.1%). Regarding academic level, there were 100 freshmen (21.6%), 114 sophomores (24.7%), 115 juniors (24.9%), and 133 seniors (28.8%). In terms of academic disciplines, 242 students were from liberal arts majors (52.4%) and 220 were from STEM (science, technology, engineering, and mathematics) majors (47.6%). Additionally, 184 participants had siblings (39.8%), whereas 278 did not (60.2%). Finally, 276 students came from urban areas (59.7%) and 186 came from rural areas (40.3%).

**Table 1:** Demographic distribution of participants.

Demographic Variables	Categories	Number of People	Percentage
Gender	Male	212	45.89
	Female	250	54.11
Major	Liberal Arts	242	52.38
	Science & Engineering	220	47.62
Only Child Status	Only Child	184	39.83
	Not Only Child	278	60.17
Place of Origin	Urban	276	59.74
	Rural	186	40.26
Year	Freshman	100	21.65
	Sophomore	114	24.68
	Junior	115	24.89
	Senior	133	28.79

### Measures

Participants completed an anonymous online questionnaire comprising demographics and a series of scales in the order presented below. The questionnaire was administered in Mandarin. The scales used in this study were previously validated in Chinese samples and administered in Mandarin [34–36]. The socio-demographic section includes six basic demographic characteristics of the participants: gender, age, major, grade level, whether they have siblings, and their place of origin.

#### Peer relationships satisfaction scale

The quality of peer relationships was measured by Peer Relationships Satisfaction Scale adapted from the PROMIS Pediatric Peer Relationship Scale [37]. The Chinese version of this scale comprises 20 items and three dimensions: interpersonal interaction (5 items), interpersonal harmony (9 items), and social emotions (6 items). An example of the items was “I was good at making friends.”Respondents rated each item on a scale ranging from "completely disagree" (1 point) to "completely agree" (5 points). The sum score of each item was calculated, with higher scores indicating better quality of peer relationships. Previous studies have established satisfactory reliability and validity regarding this scale [34]. The Cronbach alpha coefficient of this scale in this study was 0.89.

#### Core Self-Evaluations Scale (CSES)

The CSES consists of 10 items [38]. An examplar item was “I am confident I get the success I deserve.”Participants rated each item on a 5-point scale ranging from "strongly disagree" (1 point) to "strongly agree" (5 points). The sum score of each item was calculated, with higher scores indicating higher CSE. The Chinese version of CSES has satisfactory reliability and validity [35]. The Crobach alpha coefficient of this scale in this study is 0.89.

#### UCLA Loneliness Scale—Version 3 (UCLA-3)

This scale comprises 20 items, and respondents rate each item on a scale ranging from "never" (1 point) to "always" (4 points). The sum score of each item was calculated, with higher scores indicating a stronger sense of loneliness [39]. An examplar question of the scale was “How often do you feel that you lack companionship?” Scores between 20–28 represent low loneliness, 28–33 indicate mild loneliness, 33–39 suggest moderate loneliness, 39–44 signify high loneliness, and scores above 44 indicate a very high level of loneliness [36]. The Cronbach alpha coefficient of this scale in this study is 0.92.

### Statistical methods

Statistical analysis was conducted using SPSS 26.0 software. Pearson correlation analysis was first employed to explore the relationships between the perceived peer relationship quality, CSE, and loneliness. Next, the mediation effect of CSE in the relationship between perceived peer relationship quality and loneliness among university students was tested using the Bootstrap method with the SPSS plug-in "Process" developed by Hayes [40]. Independent sample t-tests were performed to compare differences in perceived peer relationship quality, CSE, and loneliness scores between different demographic groups, while one-way analysis of variance (ANOVA) was used for comparisons among multiple groups.

## Results

### Descriptive statistics

As shown in Table 2, the average score for perceived peer relationship quality among the 462 university students was 76.18 (*SD* = 10.28). The average score for CSE was 35.41 (*SD* = 7.36), and the average score for loneliness was 39.42 (*SD* = 9.84). Additionally, the majority of individuals in this group experienced a high level of loneliness, as indicated in Table 3.

**Table 2:** Descriptive statistics of scores on each scale. (*n* = 462).

	Min	Max	Mean	*SD*
Perceived peer relationship quality (20–100)	39	99	76.18	10.28
CSE (10–50)	15	50	35.41	7.36
Loneliness (20–100)	20	73	39.42	9.84

**Table 3:** Specific distribution of loneliness. (n = 462).

Loneliness Level	Low Loneliness (20–28)	Slight Loneliness (28–33)	Moderate Loneliness (33–39)	High Loneliness (39–44)	Profound Loneliness (44–80)
Frequency	40	113	84	88	137
Percentage (%)	8.7	24.5	18.2	19.0	29.7

### Correlations among the variables

There were significant negative correlations (p-value < 0.001) observed between perceived peer relationship quality and loneliness, as well as between CSE and loneliness, among university students. Additionally, there was a significant positive correlation (p-value < 0.001) between perceived peer relationship quality and CSE, as shown in Table 4.

**Table 4:** Correlation between peer relationships, CSE, and loneliness in university students.

	Peer Relationships	CSE	Loneliness
Peer Relationships	1		
CSE	0.57***	1	
Loneliness	-0.71***	-0.73***	1

### The mediating role of CSE in the relationship between peer relationships and loneliness

The Bootstrap method was employed to test for mediation effects (distinguishing whether there is a significant mediation effect based on whether the confidence interval includes 0; the absence of 0 indicates the presence of a significant mediation effect, while the presence of 0 indicates its absence). Model 4 corresponding to a simple mediation effect was chosen, with a sample size set at 5000 and a confidence interval at 95%. Gender and place of origin were used as control variables, with perceived peer relationship quality as the independent variable, CSE as the mediator, and loneliness as the dependent variable.

**Fig 2:**
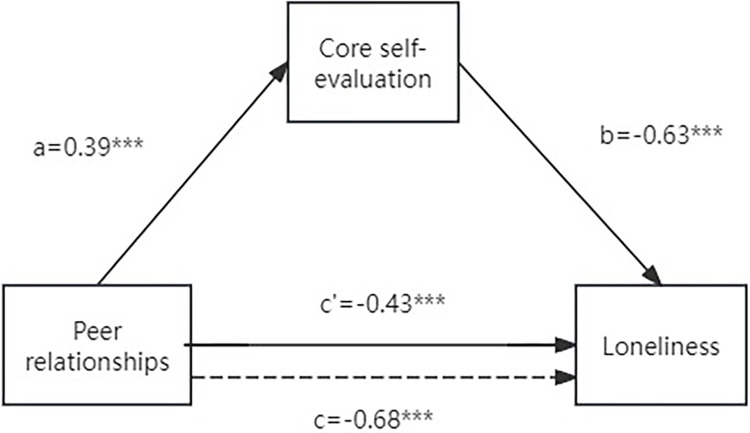
Path coefficients for mediation model. a, b, c and c’ are path coefficients representing unstandardized regression weights. The c path coefficient represents the total effect of peer relationship on the loneliness. The c-prime path coefficient refers to the direct effect of peer relationship on the loneliness. All analyzed paths were significant, *** *p* < .001.

As shown in Table 5, after controlling for gender and place of origin, perceived peer relationship quality significantly predict CSE (p < 0.001). Perceived peer relationship quality also significantly predict loneliness (p < 0.001). When the mediator variable, CSE, is included, the predictive effect of perceived peer relationship quality on loneliness diminishes but remains significant (p < 0.001). As demonstrated in Table 6, CSE plays a significant mediating role between perceived peer relationship quality and loneliness among university students (confidence interval [-0.30, -0.20] does not include 0). The mediating effect accounts for 36.23% of the total effect. The path coefficients are shown in Fig 2. The results showed that CES partially mediated the relationship between perceived peer relationship quality and loneliness because peer relationships were a significant predictor of loneliness when controlling for CES.

**Table 5:** Mediation model test of CSE in the relationship between peer relationships and loneliness in university students.

Dependent Variables	Predictor Variables	*β*	*t*	95%CI		*R^2^*	*F*
				LLCI	ULCI		
CSE	Gender	-1.55	-2.66**	-2.69	-0.40	0.33	76.48***
	Place of Origin	-0.11	-0.19	-1.26	1.04		
	Peer Relationships	0.39	13.62***	0.33	0.44		
Loneliness	Gender	0.79	1.19	-0.52	2.10	0.51	159.53***
	Place of Origin	-0.59	-0.88	-1.90	0.73		
	Peer Relationships	-0.68	-20.80***	-0.74	-0.62		
Loneliness	Gender	-0.19	-0.34	-1.29	0.91	0.66	222.35***
	Place of Origin	-0.66	-1.17	-1.75	0.44		
	Peer Relationships	-0.43	-13.42***	-0.50	-0.37		
	CSE	-0.63	-14.19***	-0.72	-0.55		

* *p* < .05, ** *p* < .01, *** *p* < .001.

**Table 6:** The mediating effect of CSE in university students between peer relationships and loneliness is decomposed.

Effects	Effect Value	Boot Standard Error	BootCI Lower Limit	BootCI Upper Limit	Relative Effect Value
Total Effect	-0.68	0.03	-0.74	-0.62	
Direct Effect	-0.43	0.03	-0.50	-0.37	63.77%
Indirect Effect	-0.25	0.03	-0.30	-0.20	36.23%

### Analysis of differences based on demographic variables

The results of the demographic differences by gender, major, sibling status, place of origin, and academic status are provided in Table 7. Statistical significance was observed for gender differences in perceived peer relationship quality, CSE, and loneliness scores (all *p*-values < 0.001). Male students exhibited higher scores in perceived peer relationship quality and CSE compared to female students, while female students reported higher levels of loneliness than their male counterparts. Differences related to being only children (i.e., single child) were also statistically significant for both perceived peer relationship quality and CSE (both *p*-values < 0.05), with only children outscoring those with siblings. Statistical significance was observed in the differences related to the students’ places of origin for perceived peer relationship quality, CSE, and loneliness scores (all *p*-values < 0.05). Urban students exhibited higher scores in both perceived peer relationship quality and CSE compared to their rural counterparts, while rural students reported higher levels of loneliness than urban students. Furthermore, there were statistically significant differences based on the students’ academic years for both perceived peer relationship quality and CSE (both *p*-values < 0.05), with sophomore (second-year) students scoring the highest and senior (fourth-year) students scoring the lowest, as shown in Table 7.

**Table 7:** Differences in peer relationships, CSE, and loneliness in university students with different demographic variables.

Demographic Variables	Category	Statistical Values	Peer Relationships *M(SD)*	CSE *M(SD)*	Loneliness *M(SD)*
Gender	Male		78.86 (9.49)	37.29 (7.45)	37.22 (9.01)
	Female		73.92 (10.39)	33.82 (6.90)	41.29 (10.14)
		*t*	5.30**	5.20***	-4.53***
		*p*	<0.001	<0.001	<0.001
		*Cohen’s d*	0.50	0.49	-0.42
Major	Humanities		76.17 (10.12)	35.34 (7.20)	39.85 (10.12)
	STEM		76.20 (10.48)	35.49 (7.54)	38.95 (9.51)
		*t*	-0.04	-0.22	0.98
		*p*	0.967	0.825	0.328
		*Cohen’s d*	-0.01	-0.02	0.09
Sibling Status	No siblings		77.97 (9.92)	36.26 (7.54)	38.46 (10.03)
	Have siblings		75.00 (10.36)	34.85 (7.19)	40.06 (9.67)
		*t*	3.07**	2.03*	-1.72
		*p*	0.002	0.043	0.087
		*Cohen’s d*	0.29	0.19	-0.16
Place of Origin	Urban		77.74 (9.90)	36.14 (7.41)	38.56 (9.75)
	Rural		73.88 (10.43)	34.33 (7.17)	40.69 (9.85)
		*t*	4.02***	2.60**	-2.30*
		*p*	<0.001	0.010	0.022
		*Cohen’s d*	0.38	0.25	-0.22
Academic Year	Freshman		77.56 (9.67)	36.15 (7.85)	39.63 (9.60)
	Sophomore		78.01 (11.05)	36.59 (7.93)	38.36 (10.76)
	Junior		76.31 (9.83)	35.35 (6.80)	38.77 (9.43)
	Senior		73.47 (9.96)	33.90 (6.72)	40.73 (9.48)
		*F*	5.01**	3.22*	1.41
		*p*	0.002	0.023	0.239
		*η^2^*	0.03	0.02	0.01

Note: *p < 0.05,**p < 0.01,***p < 0.001.

## Discussion

The findings of this study highlight the intricate interplay between perceived peer relationship quality, core self-evaluation (CSE), and loneliness among university students. The significant negative correlation between perceived peer relationship quality and loneliness aligns with prior research emphasizing the crucial role of social connections in mitigating feelings of isolation. Similarly, the negative association between CSE and loneliness underscores the importance of positive self-perception in fostering emotional well-being. Notably, the mediation analysis revealed that CSE accounted for 36.23% of the total effect of perceived peer relationship quality on loneliness, emphasizing its pivotal role in this dynamic. These results suggest that while high-quality peer relationships are essential for reducing loneliness, the internal mechanism of self-evaluation substantially influences how social interactions impact emotional outcomes.

Our study highlights the inverse relationship between university students’ feelings of loneliness and two key factors: peer relationships and CSE. Peer relationships are particularly crucial for university students, as they significantly contribute to their social adjustment and sense of belonging within the academic community. When peer relationships are fulfilling and meet students’ social expectations, they report lower levels of loneliness [41]. In contrast, students who experience unsatisfactory peer connections often struggle with emotional disconnection, exacerbating feelings of loneliness. The concept of CSE provides further insight into this dynamic. CSE, as a personality trait, shapes how individuals interpret social interactions. Students with higher CSE tend to have positive self-schemas, which leads them to focus on the more positive aspects of their social experiences. This perspective reduced their susceptibility to loneliness. On the other hand, students with lower CSE are more likely to focus on the negative aspects of social interactions, reinforcing their sense of isolation. This aligns with schema theory, which explains how personality traits affect the processing of external events [42].

The findings suggest that low CSE is associated with lower quality of peer relationships. Difficulties in communication or failure to establish strong peer connections can lead to negative cognitive patterns, such as self-deprecation, which reduce self-worth and hinder the formation of meaningful relationships [43]. In turn, students with inadequate peer support may struggle with goal achievement, face frequent failures, and experience diminished self-esteem, all of which can intensify feelings of loneliness. Moreover, while students typically seek social support, not all friendships provide the needed mutual benefit. Research shows that lower-quality peer relationships can lead to reduced self-esteem and heightened feelings of loneliness [10]. CSE plays a pivotal role in shaping students’ life experiences by influencing how they assimilate and respond to their environment [44]. For instance, students with low CSE may withdraw from social interactions due to feelings of worthlessness, while those with high CSE might experience social rejection due to perceived overconfidence. Studies have revealed curvilinear relationship between CSE and perceived social acceptance [45]. Our study shows that the quality of peer relationships positively influences CSE among university students.

These findings extend those reported in a number of other studies examining the potential contributing factors of loneliness with new evidence on the determinants and mechanisms underlying individual differences in loneliness in early adulthood [46]. When adjusting for gender and geographic origin, our analysis revealed that CSE had a significant indirect effect on the relationship between perceived peer relationship quality and loneliness experienced by university students during the pandemic. The mediation analysis highlighted two pathways through which peer relationships affected loneliness: a direct path and an indirect path via CSE. Based on Cooley’s "looking glass self" theory [47], self-concept is largely shaped through social interactions. For university students, peer relationships are the primary mode of interaction and play a crucial role in their self-understanding. Positive peer interactions typically lead to positive feedback, which, when internalized, enhances students’ perceptions of their own abilities and self-worth. This positive reinforcement of self-concept subsequently influenced their CSE. Moreover, social support, a component of which includes peer relationships, is known to positively influence CSE [48]. Viewing CSE as both a personality trait and a self-concept provides a comprehensive understanding of its role. From a personality trait perspective, it consistently affects students’ emotional responses, with higher levels of CSE often correlating with better mental health and reduced loneliness. From a self-concept standpoint, individuals with a positive self-concept actively seek and positively interpret information, contributing to better regulation of loneliness.

The study investigated perceived peer relationship quality, Core Self-Evaluation (CSE), and loneliness among Chinese university students, revealing demographic differences in these variables. Males exhibited higher quality of perceived peer relationships and CSE scores, while females reported greater loneliness. Only children scored higher in perceived peer relationships and CSE than those with siblings. Urban students showed better perceived peer relationship quality and CSE, whereas rural students experienced greater loneliness. Sophomores had the highest perceived peer relationship quality and CSE scores, whereas seniors scored the lowest. The results of the study showed that loneliness was prevalent among college students during the COVID-19 pandemic, with 18.2% experiencing moderate levels of loneliness and 29.7% feeling severely lonely. This results was aligned with previous research indicates that students experienced a moderate rise in loneliness from pre-pandemic levels to the pandemic period in Germany [49,50]. Compared to previous studies involving younger adolescents, our study showed higher loneliness levels. Earlier studies with young adults found 2.5% to 18.4% of participants reporting moderate to severe loneliness [51]. The increased loneliness observed in this study may be attributed to government-mandated lockdowns aimed at controlling viral transmission. The closure of universities due to COVID-19 has had a significant impact. Psycho-social development theory suggests that college students are at a crucial stage in forming close relationships or facing isolation. The pandemic’s restrictions severely limited social interactions, intensifying feelings of loneliness.

Previous research has shown that certain groups, such as young adults, women, individuals with lower education or income, those who are economically inactive, people living alone, and urban residents, are more likely to experience loneliness [5]. Our study also revealed significant gender differences, with female students reporting higher levels of loneliness than their male counterparts. This aligns with existing research [52]. Women, often described as more emotionally attuned, may feel lonely due to heightened sensitivity to emotional fluctuations. In contrast, men, typically characterized as more rational and less focused on their internal emotions, reported lower levels of loneliness [53]. Additionally, our analysis highlighted geographic differences, showing that students from rural areas experienced greater loneliness than their urban peers. This is consistent with previous studies and may be due to the unique challenges rural students face in adjusting to urban academic environments, which can increase their anxiety and feelings of loneliness [54]. Interestingly, we found no significant difference in loneliness between students with or without siblings, which contrasts with previous studies that found only children to experience lower levels of loneliness compared to those with siblings [55]. This suggests that sibling relationships may not be sufficient to offset the social benefits gained from peer interactions.

This study had certain limitations. First, this study is cross-sectional in nature, which limits our ability to establish a causal relationship between peer relationships, CSE, and loneliness. With ongoing changes in the COVID-19 situation and the gradual return to normalcy, university students’ social, physical, and mental conditions may evolve. Therefore, further investigation employing longitudinal study designs is warranted to unravel the temporal sequence of the relationship between peer relationships and CSE and explore the variations in students’ loneliness levels over time. In addition, our reliance on self-reported data introduces the possibility of a common-method bias. One significant limitation is the potential for social desirability bias, which can lead to an inaccurate portrayal of the participants’ actual experiences. Future studies should combine more objective or observational data to examine these relationships. Finally, the mechanisms underlying the relationship between university students’ peer relationships and feelings of loneliness require further exploration. This study examined only the mediating role of CSE. In the future, additional variables can be included to provide different perspectives for interventions targeting loneliness among university students.

## Conclusion

The quality of relationships and levels of loneliness are significantly associated with Core Self-Evaluations (CSE). Individuals with higher perceived peer relationship quality often report higher CSE, reflecting greater confidence, resilience, and a strong sense of self-worth, which can reduce feelings of loneliness. In conclusion, our findings suggest that fostering higher perceived peer relationship quality may contribute to improved CSE among university students, thereby alleviating loneliness. This dual perspective of CSE as both a stable personality trait and malleable self-concept offering opportunities for targeted interventions to address loneliness in this demographic group. This perspective highlights the potential for targeted interventions to strengthen peer relationships and establish a healthy self-concept, which ultimately leads to enhanced overall well-being.
